# Morphological analysis and stage determination of anther development in Sorghum [*Sorghum bicolor* (L.) Moench]

**DOI:** 10.1007/s00425-022-03853-y

**Published:** 2022-03-14

**Authors:** Haydee E. Laza, Harsimran Kaur-Kapoor, Zhuanguo Xin, Paxton R. Payton, Junping Chen

**Affiliations:** 1 Plant Stress and Germplasm Development, USDA-ARS, Lubbock, TX 79415 USA; 2 Department of Plant and Soil Sciences, Texas Tech University, Lubbock, TX USA

**Keywords:** Sorghum, Sorghum anther, Anther development stage, Cryo-scanning electron microscopy

## Abstract

**Main Conclusion:**

The characteristics of sorghum anthers at 18 classified developmental stages provide an important reference for future studies on sorghum reproductive biology and abiotic stress tolerance of sorghum pollen.

**Abstract:**

Sorghum (*Sorghum bicolor* L. Moench) is the fifth-most important cereal crop in the world. It has relatively high resilience to drought and high temperature stresses during vegetative growing stages comparing to other major cereal crops. However, like other cereal crops, the sensitivity of male organ to heat and drought can severely depress sorghum yield due to reduced fertility and pollination efficiency if the stress occurs at the reproductive stage. Identification of the most vulnerable stages and the genes and genetic networks that differentially regulate the abiotic stress responses during anther development are two critical prerequisites for targeted molecular trait selection and for enhanced environmentally resilient sorghum in breeding using a variety of genetic modification strategies. However, in sorghum, anther developmental stages have not been determined. The distinctive cellular characteristics associated with anther development have not been well examined. Lack of such critical information is a major obstacle in the studies of anther and pollen development in sorghum. In this study, we examined the morphological changes of sorghum anthers at cellular level during entire male organ development processes using a modified high-throughput imaging variable pressure scanning electron microscopy and traditional light microscopy methods. We divided sorghum anther development into 18 distinctive stages and provided detailed description of the morphological changes in sorghum anthers for each stage. The findings of this study will serve as an important reference for future studies focusing on sorghum physiology, reproductive biology, genetics, and genomics.

**Supplementary Information:**

The online version contains supplementary material available at 10.1007/s00425-022-03853-y.

## Introduction

Sorghum (*Sorghum bicolor* L. Moench) is the fifth-most important cereal crop in the world. It is also known for its high resilience to drought and heat stresses compared to other major cereal crops during vegetative growing stage. However, like other cereal crops, the reproductive tissues of sorghum plants are very sensitive to drought and high temperatures (Jain et al. 2007, 2010; Prasad et al. 2015; Djanaguiraman et al. 2018). High temperature and drought that occur during reproductive developmental stage cause reduction in plant fertility, failure of pollination (Jagadish 2020; Lohani et al. 2020), abortion of embryo, and shortening of the grain filling period, resulting in decrease in seed number, seed size and seed quality (Prasad et al. 2015; Chiluwal et al. 2020). Therefore, drought and heat stresses are detrimental to sorghum yield, especially as the predicted trend of climate change escalates (Hogy et al. 2013; Dingkuhn et al. 2017; van Es 2020).

Studies have shown that, among the reproductive tissues, the male tissue is most sensitive to heat stress (Giorno et al. 2013; De Storme and Geelen 2014; Prasad et al. 2015; Djanaguiraman et al. 2018). Heat stress negatively affects male reproductive organ during all stages of its development, from meiotic cell division, tapetal cell and pollen development, to pollen viability, pollen germination, and fertilization (Lohani et al. 2020; Harsant et al. 2013; Arshad et al. 2017; De Storme and Geelen 2014; Begcy et al. 2019). The resulting phenotypes observed are heat induced male sterility, reductions in pollen production and pollination efficiency (Begcy et al. 2019; Djanaguiraman et al. 2018; Harsant et al. 2013; Prasad et al. 2015). Knowledge of the (1) genes and genetic networks critical in regulating heat stress tolerance, and (2) molecular processes being affected most by heat (and other abiotic stresses) but key to yield is essential for the success in breeding enhanced, environmentally resilient sorghum.

Anther development has been morphologically studied in several plant species. In *Arabidopsis*, anther development is classified into 15 distinct stages (Sanders et al. 1999) while anther development in rice is divided into 14 stages. (Zhang et al. 2011). A recent study of wheat anthers classified anther development into 15 stages (Browne et al. 2018). In sorghum, the changes of pollen wall, tapetal orbicular wall and pollen pore development have been described in dissected anthers in early 1970s (Christensen et al. 1972; Christensen and Horner Jr^1974^). However, to date, the morphological changes throughout the entire sorghum anther developmental process have not been investigated. In addition, the developmental stages of sorghum anthers have not been determined. Lack of such critical information and standards impedes the studies on the mechanisms regulating male reproductive tissue development and their responses to abiotic stresses and on potential modification and improvement of male reproductive tissues for important agronomical traits. In this study, we examined the morphological changes at cellular level during sorghum anther development using a modified high-throughput imaging variable pressure scanning electron microscopy (VP-SEM) method (Laza et al. 2021) and traditional light microscopy methods. We divided sorghum anther development into 18 distinctive stages and provided detailed description of the morphological changes in sorghum anthers throughout the18 developmental stages. The findings in this study can be used in the studies of the regulation of genic and cytoplasmic male sterility, dissecting the mechanisms of abiotic stress responses in male reproductive tissues, as well as, in designing strategies to modify specific traits in breeding sorghums with improved agnomical traits and/or enhanced resilience to environmental stresses.

## Materials and methods

### Plant material and growing conditions

**Fig. 1:**
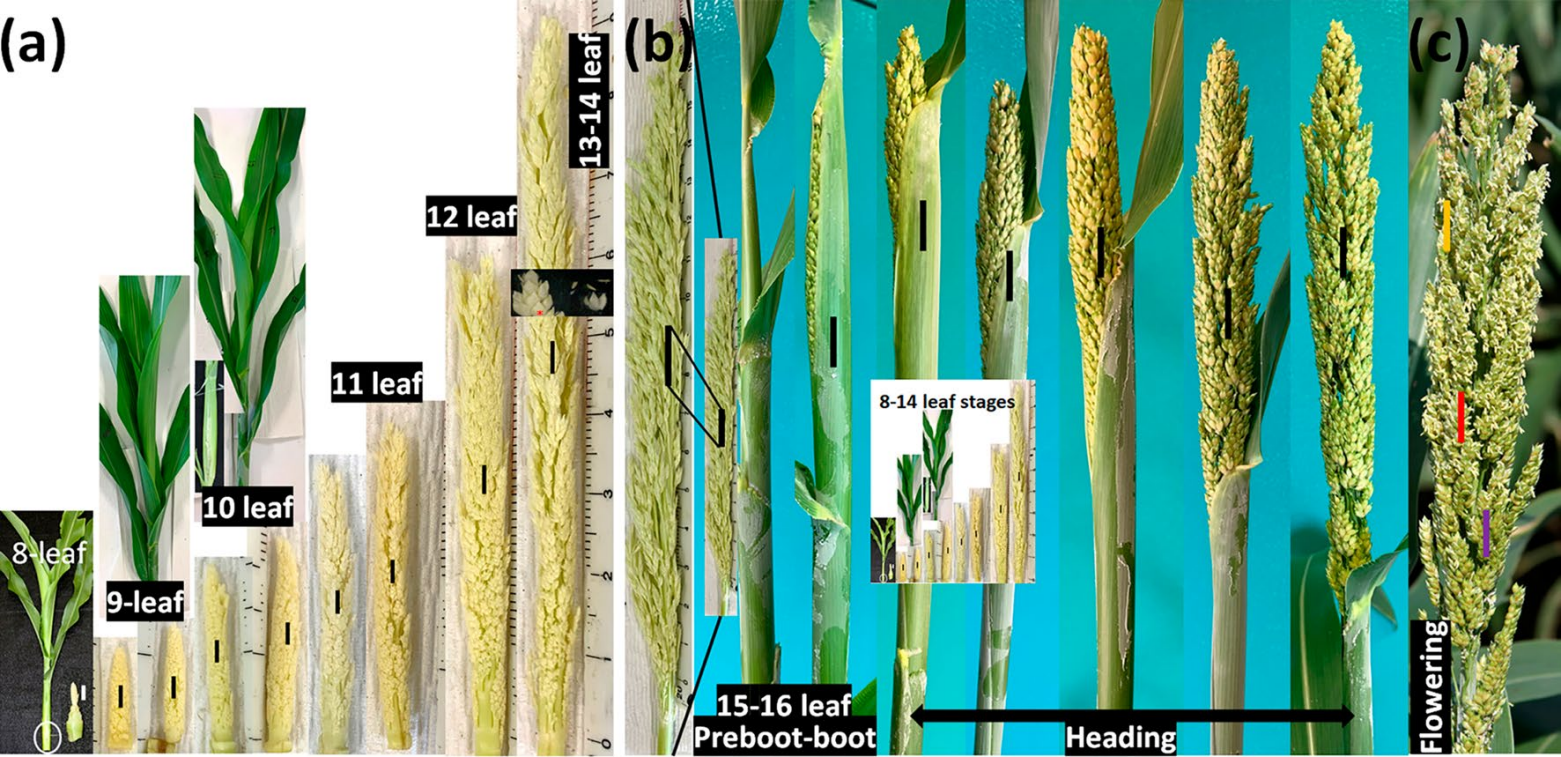
Illustration of sorghum panicle sampling for morphological and structural analysis from the 8-leaf stage to anthesis of BTx623 plants grown in greenhouse under optimal conditions. The solid bars indicate the positions where the panicle tissue samples were collected for morphological and structural analyses. **a** sorghum panicles excised from 8-leaf to 14-leaf plants; **b** sorghum panicles at different developmental stages, from pre-boot, boot, to the end of heading period. The panicle on the left were from the pre-boot sorghum; **c** sorghum panicle at flowering stage. The purple, red and yellow bars indicate the pre-pollen shed, pollen shedding, and post-pollen shed panicle sections harvested for S16, S17 and S18 pollen developmental stage analyses, respectively. The measuring scale showed in photographs are in centimeters. All panicles in (**a**) are on the same scale, whereas the plants and the insert in (**a**) are not. All panicles in (**b**), including those in the insert, also are on the same scale except for the enlarged panicle on the left. Please note that the unit measuring scale in (**b**) is much smaller than that presented in (**a**). The white circle indicates the stem area where the inflorescence was collected from 8-leaf stage sorghum plants. For 8-leaf to 14-leaf plants, their inflorescence tissues were still embedded in leaves. The panicles from these sorghum plants were collected by longitudinally cutting the leaf sheaths of the stem sections followed by carefully removal of all the leaf sheaths wrapped around the inflorescence tissues, as showed in 10-leaf plant insert in (**a**)

The experiment was conducted at USDA–ARS Cropping Systems Research Laboratory in Lubbock, TX (33° 35′ N, 101° 53′ W) and the sequenced sorghum inbred BTx623 was used in this study. The BTx623 seeds were planted in 10 L plastic pots (diameter 23 cm, H 24 cm) containing Sunshine #1 growth mix (Sun Gro Horticulture, Bellevue, WA) and thinned to 4 plants per pot at the three-leaf stage. The seeds were planted in batches every 5 days in a greenhouse for a total of four planting dates. Plants were grown under optimal conditions with temperatures set at 28 °C/23 °C, day/night, and light intensity of 400–550 µmol quanta m^−2^ s^−1^ during the day. The average day length over the sorghum growing period was about 11.5 h/12.5 h day/night. Plants were fertilized once a week with diluted Peters Excel Fertilizer 21-5-20 (N-P-K; Scotts-Sierra Horticultural Products, Marysville, OH), and the pots were rotated twice a week to ensure uniform development. The leaf positions on sorghum plants were counted from bottom-up where the first true leaf was recorded as 1-leaf and recorded on the corresponding leaf blade using a permanent marker for easy recognition (Fig. 1a). In this study, the leaf stage depicted in Fig. 1 indicates how many leaves were fully expanded on a particular plant. For example, the panicles excised from the plants with 10 fully expanded mature leaves were labeled “10-leaf” (Fig. 1a).

### Sample collection, measurement, and data analysis

To capture anther development at different plant growth stages, panicle tissues were sampled from sorghum plants at different developmental stages, starting at the seven-leaf stage until flowering at a two- to five-day interval for each batch of plantings.

Since the sorghum panicles are embedded in leaf sheath during most of vegetative growth, young sorghum plants were cut from the soil surface first and then laid carefully on a working table. The panicles from these plants were collected by longitudinally cutting open the leaf sheaths of the stem sections on opposite sides with a sharp razor blade, followed by careful removal of all the leaf sheaths wrapped around the inflorescence tissues (Fig. 1a). The excised panicles were immediately measured for the length and photographed together with a ruler (Fig. 1) followed by sample collection for analysis. In general, the central florets from the midsection of the panicle were collected, and the section size ranged from 0.3 to 1.5 cm depending on the panicle length.

**Fig. 2:**
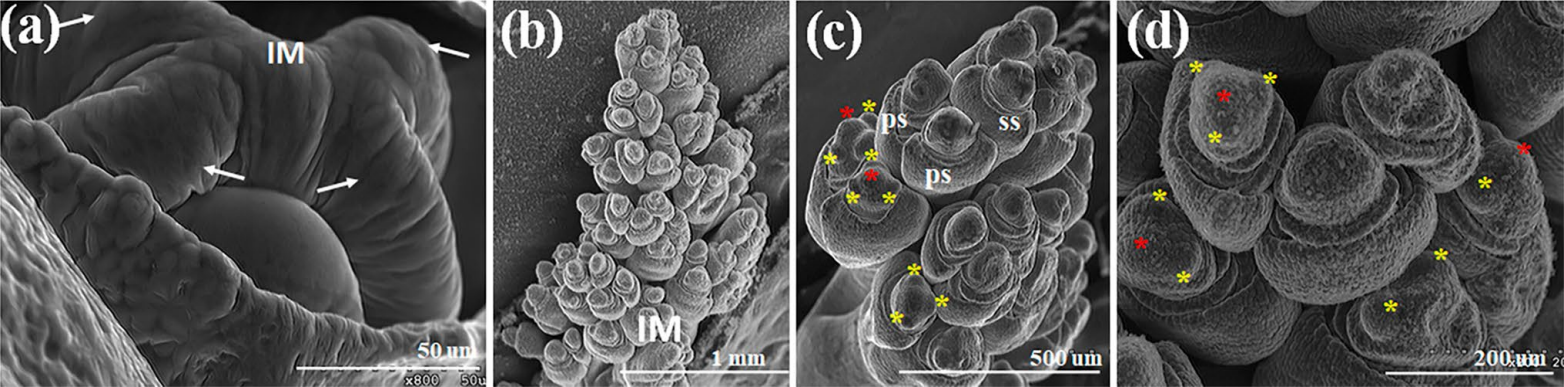
Cryo-VP-SEM and EM transverse sections images of sorghum BTx623 shoot meristem inflorescence development at the 8-leaf stage. The SEM images show the typical morphological characteristics of the (**a**) EM transverse sections inflorescence meristem (IM) at the transitional stage; (**b–d**) sessile spikelet (ss) and pedicellate spikelet (ps) during the floral transition processes and the initiation of three young stamen primordia (yellow asterisks) and pistil primordia (red asterisks). The white solid bar represents magnification of the images

A total of 200 plants, with 8 to 10 biological replicates for each development stage, were collected to study the morphological traits: the leaf number, panicle length and anther length. Selected florets in the sampled panicle tissues were dissected under a dissection microscopy to isolate the anthers for anther measurement. Also, five biological replicates (1 panicle is a unit of a replicate) for each leaf stage were collected for light and Cryo-VP-SEM microscopic analyses to examine and determine the anther developmental stages. Some of the Cryo-VP-SEM cross-sectional images were used to estimate the floret width, anther width, and anther width using Image J software (Fig. 2). Data for each trait were reported in ranges (minimum to maximum) or the mean values.

### Light microscopy and Cryo-VP-SEM microscopy

For light microscopy analysis, the panicle, flower, or anther tissues of different development stages were fixed and processed according to the procedures described (Chen et al. 2019). Briefly, the harvested tissues were fixed immediately in FAA solution (contains 95% ethanol, water, 37% formaldehyde, and acetic acid at a ratio of 50:35:10:5) followed by a series of dehydration processes at room temperature in ethanol of increasing concentrations (30 to 100% with increasing concentration by 10%), and then by 1 h incubation in 100% xylene. Dehydrated flower tissues were infiltrated, embedded in 100% Paraplast Plus (Sigma-Aldrich) at 56 to 60 °C. The embedded tissues were thinly sectioned (5 to 10 μm) using a Leica RM2125 RTS (Leica Biosystems, Buffalo Grove, IL) manual rotatory microtome, placed on a glass slide, processed, and then stained with 0.1% toluidine blue or 0.01% safranin O solution, and imaged (Chen et al. 2019). The cross sections were analyzed and photographed on an Olympus BX60 microscope equipped with an Olympus DP 80 digital camera (Olympus, Center Valley, PA, USA).

The morphological analysis of anther development on VP-SEM microscopy was performed according to the cost-effective high-throughput procedures described (Laza et al. 2021). In general, the fresh young panicles and anthers dissected from sorghum spikelet were placed on the specimen stub, cryo-immobilized in liquid nitrogen, and immediately sectioned. Sectioned tissues were quickly transferred into the SEM chamber and imaged as soon as the frozen layer melted using VP-SEM (Hitachi S-4300, Hitachi America, Tarrytown, NY) under the environmental secondary electron detector (ESDE) mode setting. The images of the freshly hydrated sorghum young inflorescences and anther sections of different developmental stages were taken at an increasing series of magnifications.

### Anther developmental stage determination

The Cryo-VP-SEM cross-sectional images were used to analyze the cellular changes and characteristics in sorghum anthers. The distinct morphological features of each anther cross section were carefully examined. The sequential morphological and structural changes from anthers of the 8-leaf stage panicle to flowering panicles were pair-wisely analyzed. The sorghum anther development stages were defined according to the distinct morphological features present in the cryo-VP-SEM cross sections and in reference to those described in other plant species including rice, maize, and wheat (Sanders et al. 1999; Zhang et al. 2011; Browne et al. 2018). The cytological analyses of anther sections under the light microscope were used for assisting stage classification and comparison with other species.

## Results

**Fig. 3:**
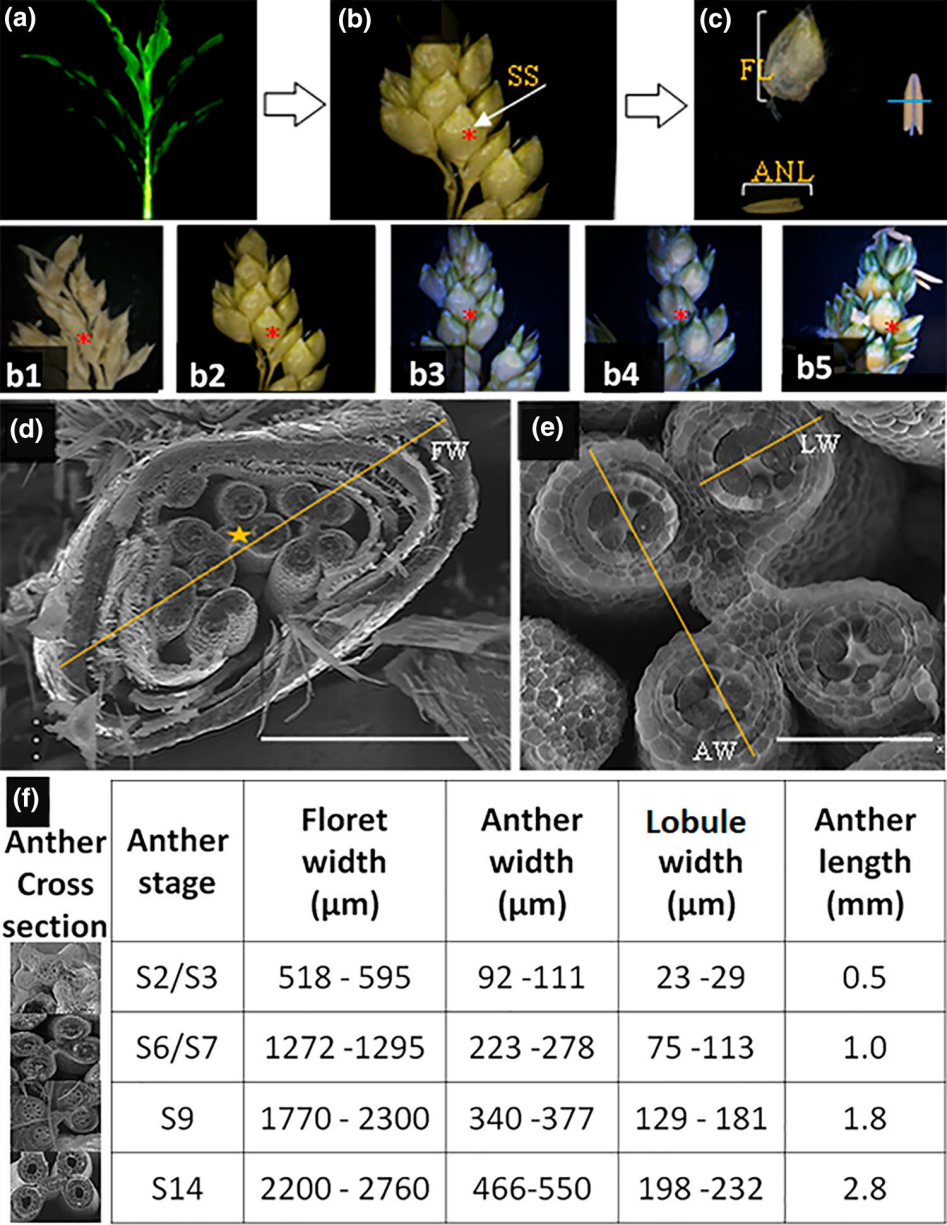
Illustration of sessile spikelets (SS) and anther trait measurements of Btx623 sorghum plants. **a** recording the leaf number of sorghum plant being analyzed; **b** photographing the harvested spikelet branches from the midsection of sorghum panicles. The b1 to b5 show the spikelet branches harvested from sorghum panicles of different developmental state where as the “*” in (**b**) indicates the central position of the selected sessile spikelet (SS); **c** measuring of the SS length (FL) and anther length (ANL) Image J software. The blue line indicates the target position (middle of floret and anther) selected for the cross-sectional analysis; **d** measuring the floret width (FW) using the Cryo-SEM cross section images of the selected sessile florets. The yellow star represents the anther selected for further measurements; **e** measuring anther width (AW), and anther lobule width (LW) using the Cryo-SEM cross section image of the selected anthers. The white bars in (**d**) and (**e**) represent 500 µm and 100 µm in length, respectively; **f** The Cryo-SEM cross section images of anthers at S1-S2, S6-S7, S9, and S14 developmental stages and the corresponding floret width, anther width, lobule width, and anther length measured

In this study, we performed detailed analysis of cryo-SEM cross sections of sorghum BTx623 inflorescence and anthers harvested at different developmental stages to capture all the morphological changes from the emergence of stamen primordia to the dehiscence and anther senescence. To capture the transition from the apical meristem to inflorescence meristem, we started to examine sorghum shoot meristem development at 6-leaf stage. Since anther development initiates from a mass of undifferentiated primordial cells at inflorescence meristem, we used Cryo-VP-SEM 3D images and transmission electron microscope cross sections to determine the initiation of stamen primordia from sorghum inflorescence meristem and compared our observation with those obtained in a previous study (Jiao et al. 2018). Our analysis showed that the shoot meristem progressed into the inflorescence developmental stage 1 around the time of sorghum plant having 8 fully expanded leaves (Fig. 1). The inflorescence meristem started to branch out from the top (Fig. 2a) followed by the formation of the triple spikelet meristem (TSM). This event occurred continuously downward along the inflorescence meristem (Fig. 2b) as plant continually grew from 8- to 9-leaf stages (Fig. 1a). The spikelet meristems on top of the inflorescence meristem started to differentiate at about 9-leaf stage (Fig. 1a) to form three stamen and one pistil primordia on each spikelet differentiated (Fig. 2b–e). The differentiation and growth of stamen and pistil (ovary) primordia on the sessile spikelets (SS) continued as plant grew to about 10-leaf stage. At this stage, the development of the inflorescence meristem in the pedicellate spikelets (PS) slowed down, leading to the arrest of the development of stamen and ovary in later stages. The SS appeared to be bigger than the PS in the inflorescence of the 11-leaf stage plants. The difference between the SS and PS became much noticeable in panicles of the 12-leaf stage plants so that SS were easily distinguished from PS (Fig. 3b1). These inflorescence developmental events observed here are consistent with those reported previously (Jiao et al. 2018).

We started to observe the anther formations in the SS in inflorescences of the 13-leaf stage plants (Fig. 1a, insert). At 13- to 14-leaf stages, the sorghum panicle started rapid expansion in length and so was the growth and development of the SS. This is also called the flag leaf stage due to the tip of the flag leaf becoming visible in the whorl. The last two or three leaves would fully expand during this period. The panicles grew rapidly in size leading to swelling of the flag leaf sheath. Sorghum plants entered boot phase at about 15- to 16-leaf stage (Fig. 1b) at which the panicles were about 50 to 75% longer than those of the 13- to 14-leaf plants (Fig. 1b). The panicles continued rapid growth and eventually pushed open the leaf sheath and up through the flag leaf collar, entering heading stage (Fig. 1b). For BTx623, heading took about 4 to 6 days. On average, anthesis started at 1 to 2 days after heading and completed in 4–5 days, about 5 to 6 cm section of the panicle bearing fresh anthers per day.

The sampling interval was shortened after sorghum plant entered pre-booting stages. Figure 1b showed a few examples of the sampling times at various panicle development stages. At flowering, we collected samples from three different sections of sorghum panicle for anther development analysis: pre-pollen shed section just beneath the flowering zone, flowering section, and the two days after flowering section (Fig. 1c). These sections represent the anthers containing mature pollen, the anthers shedding pollen, and the senesced anthers. The anther lengths at different developmental stages were provided in Table 1.

**Table 1:** Developmental stages and morphological characteristics of sorghum anther

	Stage name	Description	Anther length mm
1	Stamen primordia	The stamen primordia are observed, round in shape and contain three layers (L1, L2, L3) of undifferentiated primordial cells	Not recorded
2	Archesporial	The Epidermis (E) layer is clearly visible. The Archesporial cell (Ar) is formed. The anther primordia become more oval	0.2–0.6
3	Sporogenous	Lobule is oval in shape. The E is well differentiated, the Ar differentiated into parietal layer and the sporogenous tissues (Sp). Connective (C) and Vascular region begin to form. The 4 lobules begin to separate with a defined stomium region (StR)	0.5–0.8
4	3-layer cell wall	Lobule is round in shape with a well differentiated three anther wall layers: E, Endothecium (En) and Middle Layer (ML). Tapetum (T) begins to develop and the Sp cells are still present but more differentiated. Connective and Vascular tissues are more developed	0.5–0.9
5	Pre-callose	Tapetal layer (T) is well-defined and the Sp begins to differentiate into Microspore Mother Cells (MMC)	0.65–1.0
6	Central callose	The meiotic cells (MC), consisting of the MMC surrounding by callose are tightly close and located in the center of the lobule (6a). The cell division and callose accumulation continue and the MC are still located in the center. The tapetal cells are more developed (6b)	0.85–1.25
7	Meiotic	The meiotic cells derived from MMC meiosis move away from the center of lobule toward the wall of tapetum, leaving the center empty. Tapetal cells continue growing in size and vacuolation. The individual cells are rectangular in shape	1.0–1.4
8	Tetrad	Meiotic processes are completed. The MC breaks away from the tapetum and form the tetrads surrounded by callose wall. The middle layer is crushed	1.3–1.8
9	Young microspore	The callose surrounding the tetrads degrades and releases the young microspores (Msp) within the lobule and the tapetal cells are at their biggest size and change from rectangular to hill in shape (9a). Middle layer becomes almost invisible. At the end of this stage, the Msp are peripherally located along to the tapetal cells and the tapetum starts to degenerate (9b)	1.4–2.0
10	Final hydrated	ML is completely degraded. The tapetum layer is significantly thinner. The Msp vacuolate, increase in size and is wedge-shaped. This is the last hydrated stage	1.6–2.2
11	Dehydration initiation	The dehydration process is initiated from the center of the locule and progresses toward the tapetum. The tapetum continues its degradation	1.8–2.5
12	Vacuolate microspore	Msp vacuolate, become much larger and spherical and arrange along the T. The locule is completely dehydrated, forming an air-filled locular cavity. The T is mostly degraded and becomes uneven	1.9–2.6
13	Falcate microspore	Msp begin to dehydrate and become falcate in shape. T is completely degraded and only residues of the T are present	2.0–2.7
14	Vacuolate pollen	The Msp grow larger, become multinucleate as a result of pollen mitotic divisions, and start accumulating starch in the pollen grains (PG). The young PG is surrounded by the exine layer	2.3–3.0
15	Nucleate pollen	The PG become round in shape and filled with starch. The septum begins to degenerate and becomes thinner	2.6–3.1
16	Bilocular	The septal region degrades between the two upper and lower locules and the anther becomes bilocular	2.6–3.1
17	Dehiscence	The epidermal cell at stomium region (StR) degrades, causing the opening of locules and releases of the pollen grains. The En degrades and the E becomes thinner	2.6–3.1
18	Senescence	All PG are released, and the anther wall degrades, anther senesced	Not recorded

**Fig. 4:**
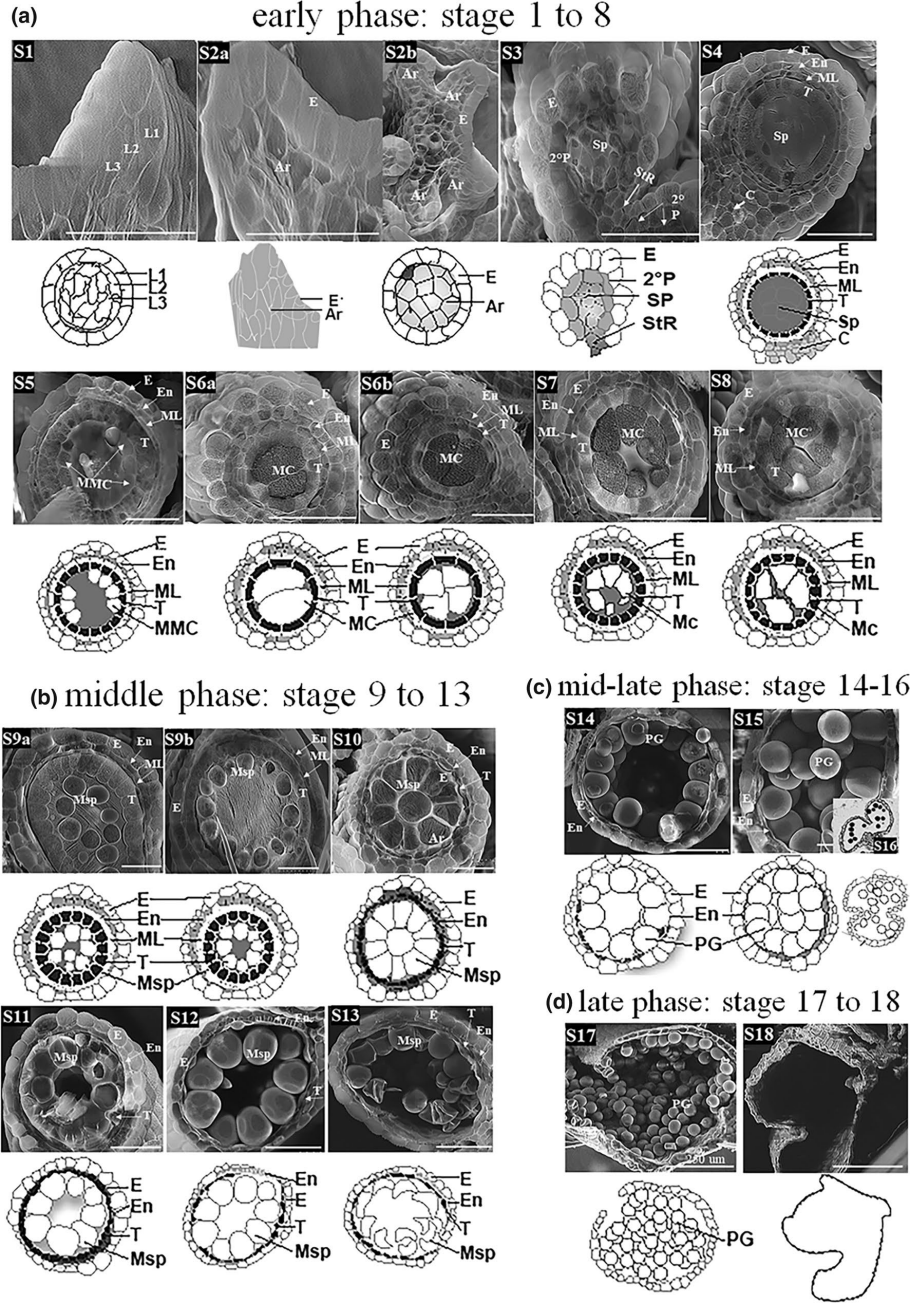
Sorghum BTx623 anther developmental stages. The 18-anther developmental stages determined by Cryo-VP-SEM transverse section analysis. The diagrams below the SEM images signify the main morphological features at each of the 18 stages and illustrate the main differences among stages. The S1-S8, S9-S13, S14-S16, and S17-S18 represent the early, middle, mid-late, and late phases of anther development, respectively. *Ar* Archesporial cell, *C* connective, *E* epidermis, *En* endothecium, *L1* 1st Cell layer, *L2* 2nd Cell Layer, *L3* 3rd Cell layer, *ML* middle layer, *MC* meiotic cells, *MMC* microspore mother cell, *Msp* microspores, *Sp* Sporogenous Tissue, *ST* stomium, *StR* stomium region, *T* tapetum, *Td* tetrads, *PG* pollen grains, *Sp* sporogenous tissues, *V* vascular bundle

In this study, anthers in sessile spikelets of harvested samples (Fig. 3b b1–b5) were used for detailed morphological characterization under different type microscopies. The Cryo-VP-SEM cross-sectional images were examined to identify morphological features of anthers collected from inflorescence sections of different developmental stages (as showed in Fig. 1). We also performed pairwise morphological feature comparison between anthers started from youngest (13-leaf) to oldest (flowering) according to plant development stages (Fig. 1). Based on the unique morphological features identified, we classified the anther development into 18 stages from the stamen primordia emergence to anther senescence (Table 1 and Fig. 4). The classification of anther development described in *Arabidopsis*, rice, maize, and wheat (Sanders et al. 1999; Zhang et al. 2011; Browne et al. 2018; Tsou et al. 2015) was used to define the stages of sorghum anther developments. The cytological analyses of anther sections under the light microscope were used for assisting stage classification (Fig. S1).

The details of cellular structural changes during the progressive anther development are showed in Fig. 4. All image photos in Fig. 4 were cross sections of sorghum anther at different developmental stages except for the images for stage 1 and 2 (S1 and S2a), which were longitudinal sections of anther primordia. The diagram beneath each SEM image signified the main morphological features at each stage and illustrated the main feature differences among the 18 stages. During very early phase of the anther development, the anther primordia were formed from differentiated inflorescence meristem cell and underwent rapid cell division. At stage 1 (S1), the round-shaped stamen primordia could be observed on top of the spikelet (Fig. 2c–d, S1). The longitudinal section of anther primordia showed 3 distinct layers of the undifferentiated cells, L1, L2 and L3, (Fig. 4a) and was round (Fig. 3a). At stage 2 (S2), the archesporial phase (Fig. 4a), a layer of epidermal cells derived from L1 layer cell divisions became clearly visible. The archesporial cells derived from L2 layer cell division were formed. The stamen primordia became more oval in shape (Fig. 4a, S2b). The archesporial cells continued to divide and differentiate to form the 1° and then 2° parietal layer and central sporogenous tissue at stage 3 (S3), sporogenous stage (Fig. 4a). The connective cells initiated from L3 layer cell division. At the end of stage 3, secondary parietal layer and the sporogenous cells became clearly visible at the four corners of the anther primordia and the 4 lobules began to separate with a defined stomium region (StR). The lobules became oval in shape and the epidermal layer was well differentiated and the cells were generally larger than other cell types (Fig. 4a). The connective tissue and vascular region began to form.

**Fig. 5:**
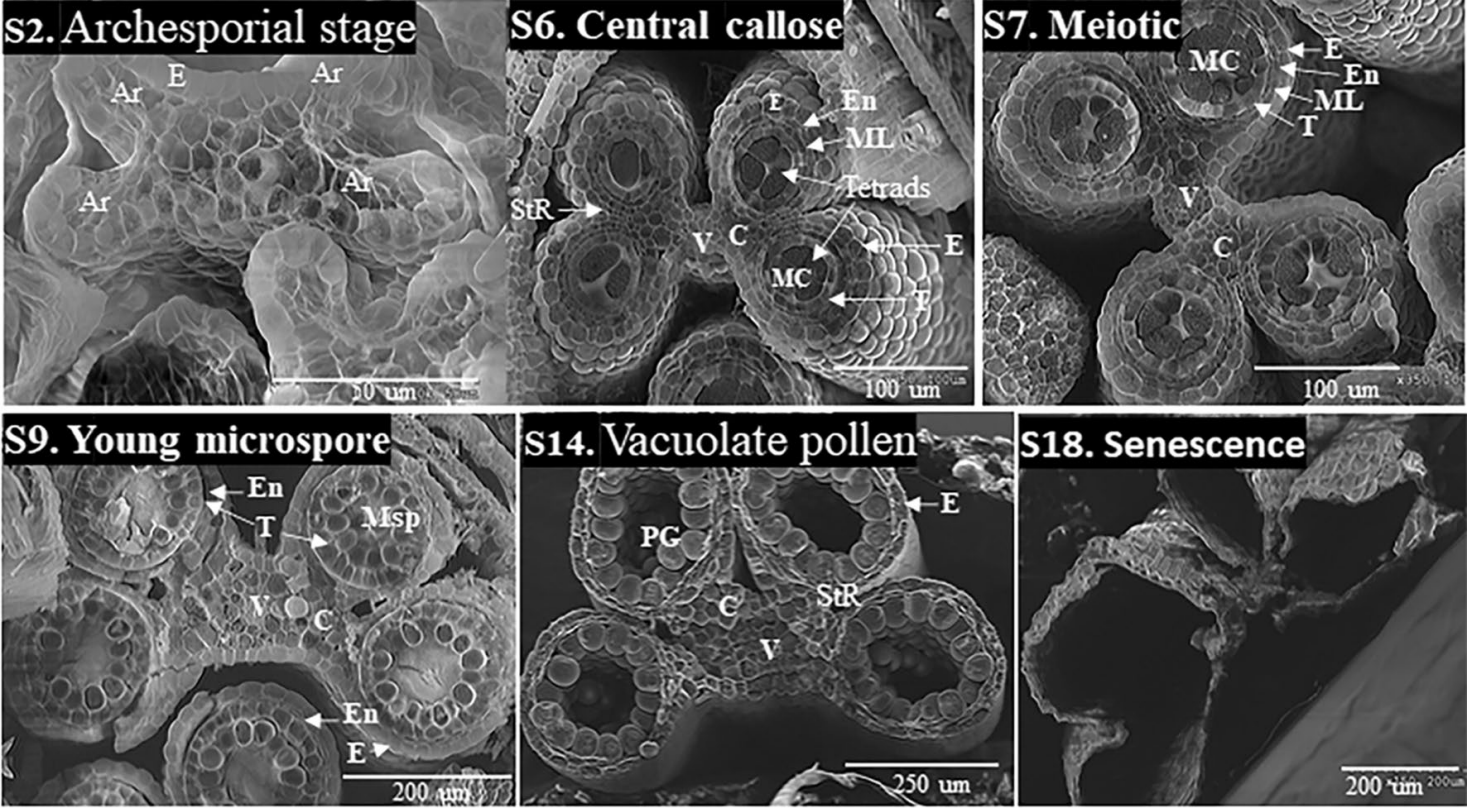
Cryo-VP-SEM images of the transverse sections of sorghum BTx623 whole anther at selected developmental stages. *Ar* Archesporial cells, *C* connective tissue, *E* epidermis, *En* endothecium, *MC* meiotic cells, *ML* middle layer, *Msp* microspores, *StR* stomium region, *T* tapetum, *PG* pollen grains, *V* vascular bundle. White solid bar represents fold of magnifications

At stage 4 (S4), the lobule became round in shape with a well differentiated three outer layers of the anther wall: the epidermis, endothecium, and middle layer (Fig. 4a). The synthesis of the tapetal cell began to form the tapetum while the sporogenous cells became more differentiated. Connective and vascular tissues were more developed. At stage 5 (S5), the tapetal layer was well-defined and becoming more differentiated (Fig. 4a). At the end of this stage, the size of tapetal cells became much larger and sporogenous cells started to differentiate into the microspore mother cell (Fig. 4a). At stage 6 (S6), the callose began to accumulate in the center of the locules and cell division continues (Fig. 4a, s1). The meiotic cells, consisting of the microspore mother cell surrounded by callose were tightly located in the center of the lobule (S6a), and the tapetal cells became more developed and started to become vacuolated (S6b) at the end of stage 6 (Figs. 4a, 5). At stage 7 (S7), the microspore mother cell began the process of meiosis to form the meiotic microspore cells, which moved toward tapetum wall from lobule center (Figs. 4a, 5, and s1). The tapetal cells continued to grow in size and became more vacuolated and rectangular in shape (Figs. 4a, 5). The anther began to more rapid increases in size from this stage forward. The meiotic processes were completed at stage 8 (S8). The meiotic microspores eventually broke away from the tapetum to form the tetrads surrounded by callose wall while the middle layer started to degenerate (Fig. 4a).

At stage 9 (S9), the callose surrounding the tetrads degraded to releases the young microspores within the lobule (Figs. 4b, 5). The vacuolated tapetal cells were at their largest (S9a) and changed from rectangular to hill-shaped (S9a). The middle layer degraded and became almost invisible. At the end of this stage (S9b), the tapetum started to degenerate and tapetal cells became denser. The free peripheral haploid microspores started to vacuolate and move toward the tapetum (Figs. 4, 5). At stage 10 (S10), the middle layer was completely degraded and the tapetum layer was significantly reduced (Fig. 4b). The microspores continued to vacuolate and increase significantly in size (Fig. 4b). This was the last hydrated stage where liquid filled the lobular central cavity of sorghum anther (Fig. 1k). The transition from hydrated to dehydrated lobular central cavity began at stage 11 (S11). The process started from the lobular center and progressed toward the tapetum. At the same time, the microspores continued to vacuolate and increase in size, and became spherical in shape (Fig. 4b). At the end of S11, the center of anther lobule became an air-filled cavity while the space surrounding microspores alongside the largely degraded tapetum remained hydrated (Fig. 4b). As the microspores continued to grow, most of them contained a single large vacuole and became spherical and larger at stage 12 (S12, Fig. 4b). In the meantime, tapetal layer was mostly degraded and became very thin and uneven (Fig. 4b). By the end of this stage, the dehydration process within the locule cavity was complete and, for the first time, filled with air (Fig. 4b). At stage 13 (S13), the large microspores began to dehydrate and became falcate in shape as the vacuolated microspores dehydrate (Figs. 4b, s1). The tapetum was completely degraded and become almost invisible (Fig. 4b).

At stage 14 (S14), the microspores grew larger and began mitotic divisions to become multinucleate. The microspores started to accumulate starch at S14 and turned into young pollen grains surrounded by the exine layer (Figs. 4c, 5). At stage 15 (S15), pollen grain was round and filled with starch (Fig. 4c, s1) and the septum began to degenerate (Fig. s1, arrow). As pollen grain matured at stage 16 (S16), the septum region connecting the upper and lower locules degraded and the anther became bilocular (Fig. 4c insert, s1). While the endothecium degraded completely at stage 17 (S17), the anther epidermis became thinner and the epidermal cell at stomium region degraded, causing the opening of locules followed by the release of the mature pollen grains (Fig. 4d). After releasing the pollen grains, the epidermis of the anther wall degraded (Fig. 4d), the anther underwent senescence at stage 18 (S18) and eventually fell from sorghum panicle. However, the bilocular anther phenotype remained visible at both S17 and S18 (Fig. 4d, s1).

According to the commonalities and differences of cellular activities occurring during anther development, we assorted the 18 stages into 4 phases: early, middle, mid-late, and late (Fig. 4). At the early phase (Fig. 4a), sorghum inflorescence meristem first underwent tissue differentiation, rapid cell division and differentiate processes to form 4 well-defined lobules with 4 distinct anther wall layers (S1–S4); this was followed by a series of meiotic activities of microspore mother cells leading to the formation of haploid tetrads (S5–S8). The middle phase (S9–S13) was a series of microspore vacuolation processes (Fig. 4b). It began with the complete degradation of callose to form free microspores (S9) and ended with dehydration of the microspores (S13). During this phase, the tapetal cells degraded leading to the disappearance of tapetum at the end (Fig. 4b). In the meantime, the microspores vacuolated and increased in size progressively (S10-S12) while they situated peripherally along to the tapetum wall (S9–S13). The transition of hydrated lobule to air-filled lobule cavity took place in the middle of this phase (S11), whereas the dehydration of vacuolated microspore occurred at the end (S13). The middle–late phase (S14–S16) was the phase where microspores developed into mature pollen and the anthers became bilocular (Fig. 4c). During this phase, starch continued to accumulate in pollen grain and anther growth reaching its maximum length while the septum between the pair of locules degraded. The late phase (Fig. 4d) included degradation of the epidermal cell at stomium region (StR) leading to the opening of locules and mature pollen grain release (S17) and anther senescence (S18).

## Discussion

The present study reports the classification of 18 stages in sorghum anther developments and the morphological characteristics associated with each stage (Table 1; Fig. 4). This is the first comprehensive study reporting the detailed descriptions of anther developmental stages in sorghum. Information presented here provides a baseline for anther development and can serve as an important reference for future studies focusing on sorghum physiology, reproductive biology, genetics, genomics studies as well as studies aimed at enhancing abiotic stress tolerance of sorghum male reproductive tissues.

The progression in structural changes observed in cryo-VP-SEM cross sections was utilized in the determination of the 18-anther developmental stages in BTx623 in this study. The main criteria include but not limit to: the presence or degradation of a given tissue and cell type, size and shape of cellular structures, and the transition between the liquid and gas filled stages. The cytological study performed by light microscopy at key stages (Figs. s1, s2) was used to assist the stage determination and in comparisons with those reported in plants (Sanders et al. 1999; Zhang et al. 2011; Tsou et al. 2015; Browne et al. 2018; Christensen and Horner Jr. 1974; Christensen et al. 1972). Stage names used in this report (Table 1) correspond largely to the nomenclatures described in wheat, rice, and *Arabidopsis* (Sanders et al. 1999; Zhang et al. 2011; Tsou et al. 2015; Browne et al. 2018).

Anther development processes have been morphologically examined and stages determined in *Arabidopsis* (15 stages), rice (14 stages) and wheat (15 stages) (Sanders et al. 1999; Zhang et al. 2011; Browne et al. 2018). In this study, the sorghum anther developmental process exhibited both similarities and differences with other plant species. We have found that later stages of anther development are much alike across a broad range of different plant species while the early stages vary somewhat among different species (Sanders et al. 1999; Zhang et al. 2011; Ekici and Dane 2012; Wang et al. 2012; Xu et al. 2014; Tsou et al. 2015; Browne et al. 2018; Chaban et al. 2020). The anther development in sorghum resembles mostly to those of maize anthers (Wang et al. 2012; Moon et al. 2013; Tsou et al. 2015), followed by those in wheat and rice (Zhang et al. 2011; Browne et al. 2018). In sorghum, the two upper and lower locules from each theca in sorghum anther are separated and the septum and stomium regions are well differentiated (Fig. 5S2). This feature is consistent with those reported in other grasses including maize (Wang et al. 2012; Moon et al. 2013), wheat (Browne et al. 2018) and rice (Zhang et al. 2011), but differs with that reported in cotton anther (Xu et al. 2014), suggesting that these features may be conserved across the grass family. Nevertheless, the timing of the “lobule formation” in sorghum anther appears to differ from those reported in wheat and rice. The formation of 4 lobules at the 4 corners of anther occurs at about stage 4 in wheat and rice (Zhang et al. 2011; Browne et al. 2018) as well as in *Arabidopsis* (Sanders et al. 1999) while in sorghum and maize, it occurs at a much earlier stage (Figs. 2-S2, and 4a) prior to the anther wall differentiation (Wang et al. 2012; Moon et al. 2013).

Most anther developmental studies were conducted using traditional light (wheat, *Arabidopsis*, rice) and/or electron microscopy (maize and sorghum) methods (Sanders et al. 1999; Zhang et al. 2011; Wang et al. 2012; Moon et al. 2013; Browne et al. 2018). Preparing anther tissues for these observations commonly consists of plastic embedding and a series of chemical processes, leading to the completely removal of intercellular liquid from tissues and therefore, unable to exam the hydration status of anther at set developmental stages. The newly modified Cryo-VP-SEM method (Laza et al. 2021) used in this study eliminated the lengthy tissue preparation processes in various chemicals and allowed us to examine sorghum anther development in its most natural state, and enabled us to compare our results with the existing literature and incorporate new findings regarding the hydration status in space filling in lobular cavities and transitional stages. Our observations show that lobules in sorghum anthers are fully hydrated S1 to S10 (Fig. 4a–b). The transition of hydrated anthers to dehydrated anthers occurs at S11 while the lobules of S12 to S18 anther are air-filled (Fig. 4b–c). The results are closely correlated with Cryo-SEM observations reported in maize (Tsou et al. 2015). Therefore, we have incorporated hydration status into stage determination in sorghum anther development (Table 1).

## Conclusion

Sorghum anther development was classified into 18 stages. The distinctive morphological features associated with each stage and the cellular structure changes over the pollen development were examined in detail. The classification of sorghum anther development provides a reference for studying reproductive biology and abiotic stress tolerance of male reproductive tissues. The findings on this study can be used in the studies of the regulation of genic and cytoplasmic male sterility, in dissecting the mechanisms of heat stress responses in male reproductive tissues and identifying the stages most vulnerable to high temperatures, as well as, in designing strategies to modify specific traits in breeding sorghums with improved agronomical traits and/or enhanced resilience to environmental stresses.

### *Authors contribution statements*

HEL contributed to the LM and Cryo-VP-SEM analyses. JC and HEL concerned this research project and conducted the experiments. HEL and HKK analyzed the LM and SEM images. HKK, PP and ZX assisted with sample collection and sample processing. JC and HEL drafted the manuscript. All authors reviewed the manuscript.

## Supplementary Information

Below is the link to the electronic supplementary material.

Supplementary file1 (TIF 6256 KB) Figure S1 Light microscopy transverse sections of sorghum BTx623 whole anther at selected developmental stages as indicated on the cytological images.

Supplementary file2 (TIF 2699 KB) Figure S2 Cryo-VP-SEM and LM transverse sections of sorghum BTx623 young flower at selected developmental stages; (a) stage 6; (b-c) stage 8. White solid bar represents: (a)=500um, (b and c) =1mm. Images a-c were examined under a variable transmission electron microscope. Images d-i were taken using a light microscope.

## Data Availability

All data analyzed during this study are included in this published article and its supplementary information file.

## Abbreviation

VP-SEM: Variable pressure scanning electron microscopy
